# A Case of Primary Leiomyoma of the Chest Wall Showing High Uptake on FDG-PET

**DOI:** 10.70352/scrj.cr.26-0403

**Published:** 2026-10-10

**Authors:** Takuzo Sakaguchi, Mamoru Takahashi, Shunji Cho, Tsuyoshi Shoji, Hiromichi Katakura

**Affiliations:** Department of Thoracic Surgery, Japanese Red Cross Otsu Hospital, Otsu, Shiga, Japan

**Keywords:** leiomyoma, chest wall tumor, FDG-PET, mesothelioma, differential diagnosis

## Abstract

**INTRODUCTION:**

Primary leiomyoma of the chest wall is a rare entity. We report a case of primary chest wall leiomyoma with intense uptake on fluorodeoxyglucose PET (FDG-PET), requiring differentiation from malignancy.

**CASE PRESENTATION:**

An 82-year-old man with a history of asbestos exposure underwent annual chest CT surveillance. A 19-mm nodule was newly identified on the right chest wall and had not been present on CT the previous year. Although the lesion had well-defined margins on CT, FDG-PET demonstrated intense uptake with a maximum standardized uptake value of 11.8, raising suspicion for malignancy. Surgical resection was performed for diagnostic and therapeutic purposes. Histopathological examination revealed fascicular proliferation of spindle-shaped cells. Immunohistochemical analysis suggested a smooth muscle tumor, and retention of BRCA1-associated protein 1 and methylthioadenosine phosphorylase excluded mesothelioma. Based on these findings, the tumor was diagnosed as a primary leiomyoma of the chest wall.

**CONCLUSIONS:**

Primary leiomyomas of the chest wall are rare and may show high FDG uptake on PET imaging, making differentiation from malignant tumors challenging. A comprehensive evaluation incorporating imaging, intraoperative findings, and pathological assessment is essential for accurate diagnosis.

## Abbreviations

α-SMA
: alpha-smooth muscle actin
ALK
: anaplastic lymphoma kinase
BAP1
: BRCA1-associated protein 1
CD34
: cluster of differentiation 34
FDG-PET
: 2-deoxy-2-[^18^F] fluoro-D-glucose PET
H&E
: hematoxylin and eosin
IHC
: immunohistochemical
MTAP
: methylthioadenosine phosphorylase
STAT6
: signal transducer and activator of transcription 6
SUVmax
: maximum standardized uptake value

## INTRODUCTION

Leiomyomas are benign tumors that commonly arise in organs containing smooth muscle, such as the uterus and gastrointestinal tract. Primary leiomyomas of the chest wall are extremely rare. Most chest wall tumors are metastatic lesions, malignant mesotheliomas, schwannomas, or fibrous tumors, and reports of leiomyomas arising in the chest wall are scarce. Herein, we report a rare case of primary leiomyoma of the chest wall and review the relevant literature.

## CASE PRESENTATION

The patient was an 82-year-old man with a history of asbestos exposure who underwent annual follow-up chest CT. A new, well-defined, 19-mm nodule was identified in the right chest wall that had not been present on the previous year’s CT. Contrast-enhanced CT showed heterogeneous enhancement without evidence of invasion into surrounding tissues (**Fig. 1A** and **1B**). FDG-PET revealed intense uptake in the lesion, with an SUVmax of 9.5 in the early phase and 11.8 in the delayed phase (**Fig. 1C**). Considering the high FDG uptake, malignancy could not be ruled out. Video-assisted thoracoscopic surgery was performed under general anesthesia for both diagnostic and therapeutic purposes. Because the patient was 82 years old, preserving postoperative ADL was considered an important surgical objective. Preoperatively, the patient was informed that if the final pathological diagnosis demonstrated malignancy or infiltrative growth, additional chest wall resection including the involved rib would be considered as a second-stage procedure. The lesion was located in the third intercostal space. It protruded into the thoracic cavity from the subpleural region. No rib invasion was observed, and the lesion had clear borders. Consequently, the lesion was resected along with portions of the pleura and intercostal muscles while preserving the rib (**Fig. 1D**). The resected specimen revealed a well-circumscribed solid tumor (**Fig. 2A**). The intraoperative frozen section diagnosis was a “spindle cell tumor.” Based on the frozen section and intraoperative findings, extended chest wall resection including the rib was deferred. Additional chest wall resection was planned only if the final pathological examination demonstrated infiltrative growth or malignancy. Histologically, spindle cells with mild atypia proliferated in fascicular and interlacing patterns (**Fig. 2B**). No mitotic figures were observed. The Ki-67 labeling index, evaluated in hotspot areas, was up to 15%. Overall, the proliferative activity was low, and no significant cellular atypia was observed. The interface between the tumor and surrounding skeletal muscle and connective tissue was clear, with no evidence of infiltrative growth.

**Fig. 1:**
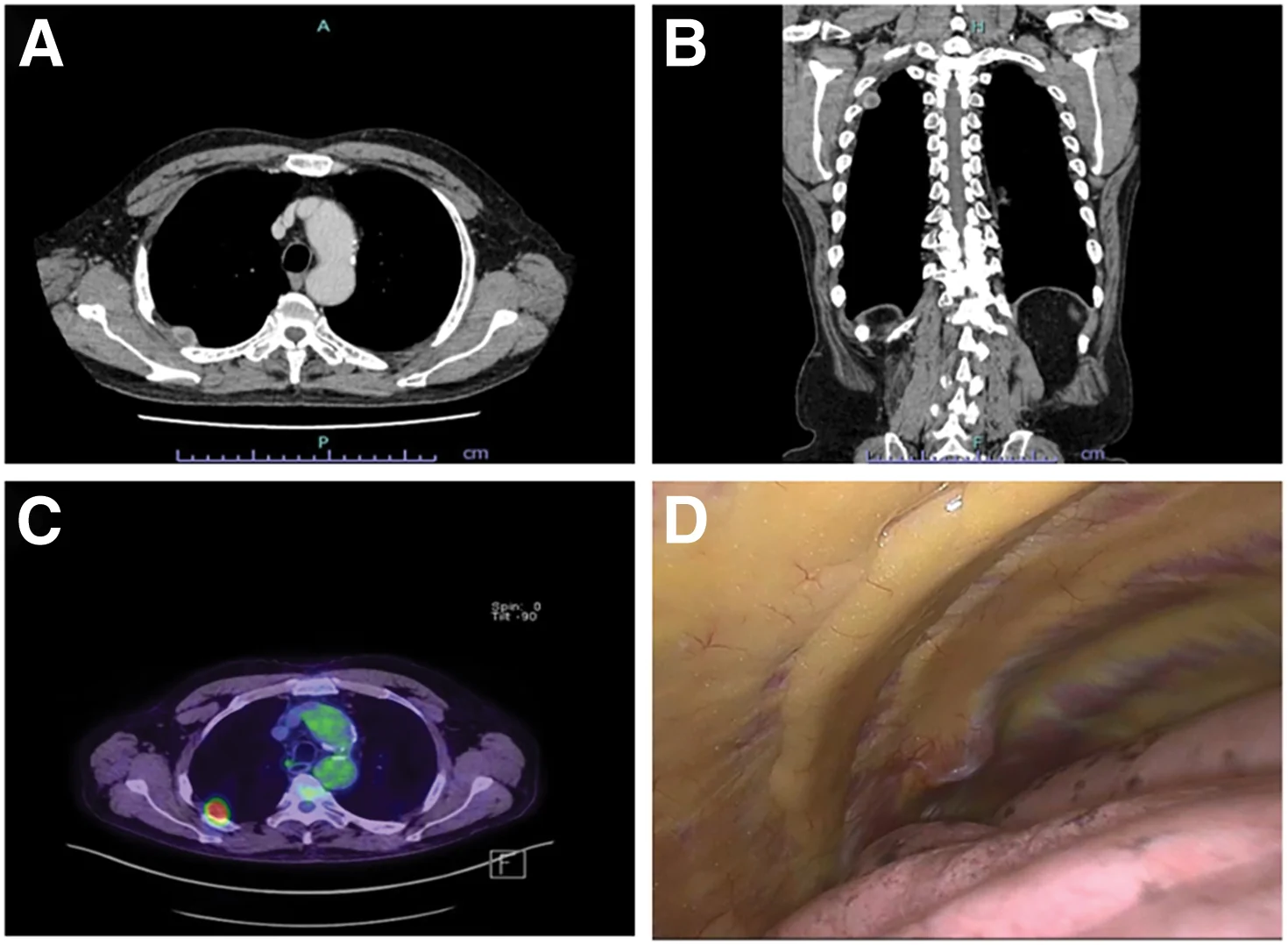
Imaging and intraoperative findings. (**A**) Contrast-enhanced CT demonstrates a well-defined nodule in the right chest wall. (**B**) Coronal CT image shows the lesion beneath the pleura without evidence of invasion into surrounding structures. (**C**) FDG-PET reveals intense uptake in the lesion (SUVmax 9.5 in the early phase and 11.8 in the delayed phase). (**D**) Intraoperative findings show a tumor protruding from the parietal pleura without rib invasion. FDG-PET, 2-deoxy-2-[^18^F] fluoro-D-glucose PET; SUVmax, maximum standardized uptake value

**Fig. 2:**
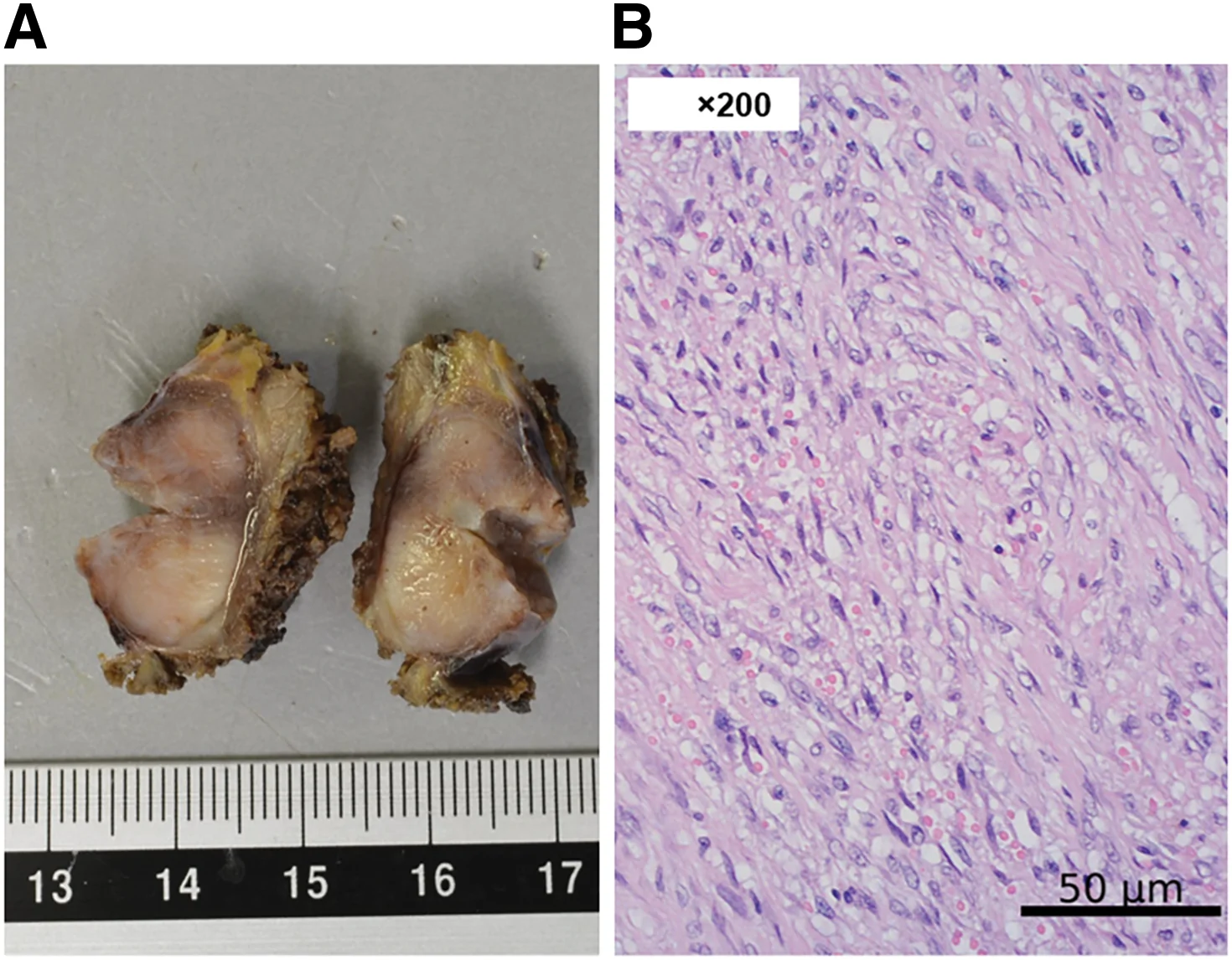
Gross and histological findings (H&E staining) of the resected specimen. (**A**) The cut surface shows a well-circumscribed, solid, whitish tumor beneath the pleura without evidence of invasion into surrounding tissues. (**B**) Spindle-shaped cells proliferate in fascicles without significant atypia or mitotic figures (×200). H&E, hematoxylin and eosin

Immunohistochemistry showed positivity for α-SMA and h-caldesmon, suggesting a smooth muscle tumor, whereas desmin was negative. Representative IHC findings are shown in **Fig. 3**. For differential diagnosis, CD34 and STAT6 were negative, ruling out a solitary fibrous tumor. β-Catenin was positive only in the cytoplasm, without nuclear expression, thereby ruling out desmoid-type fibromatosis. Furthermore, S100 was negative, ruling out schwannoma, and c-kit and ALK were also negative, excluding gastrointestinal stromal tumors and inflammatory myofibroblastic tumors, respectively. Pankeratin, cytokeratin 7, and calretinin were negative, indicating no evidence of mesothelial tumors. Given the history of asbestos exposure, BAP1 and MTAP staining were performed to exclude malignant mesothelioma. Both showed retained expression, thereby ruling out mesothelioma. Based on the morphological and IHC findings, the lesion was diagnosed as a primary leiomyoma of the chest wall. The postoperative course was uneventful. No recurrence or metastasis has been observed during 1 year of follow-up.

**Fig. 3:**
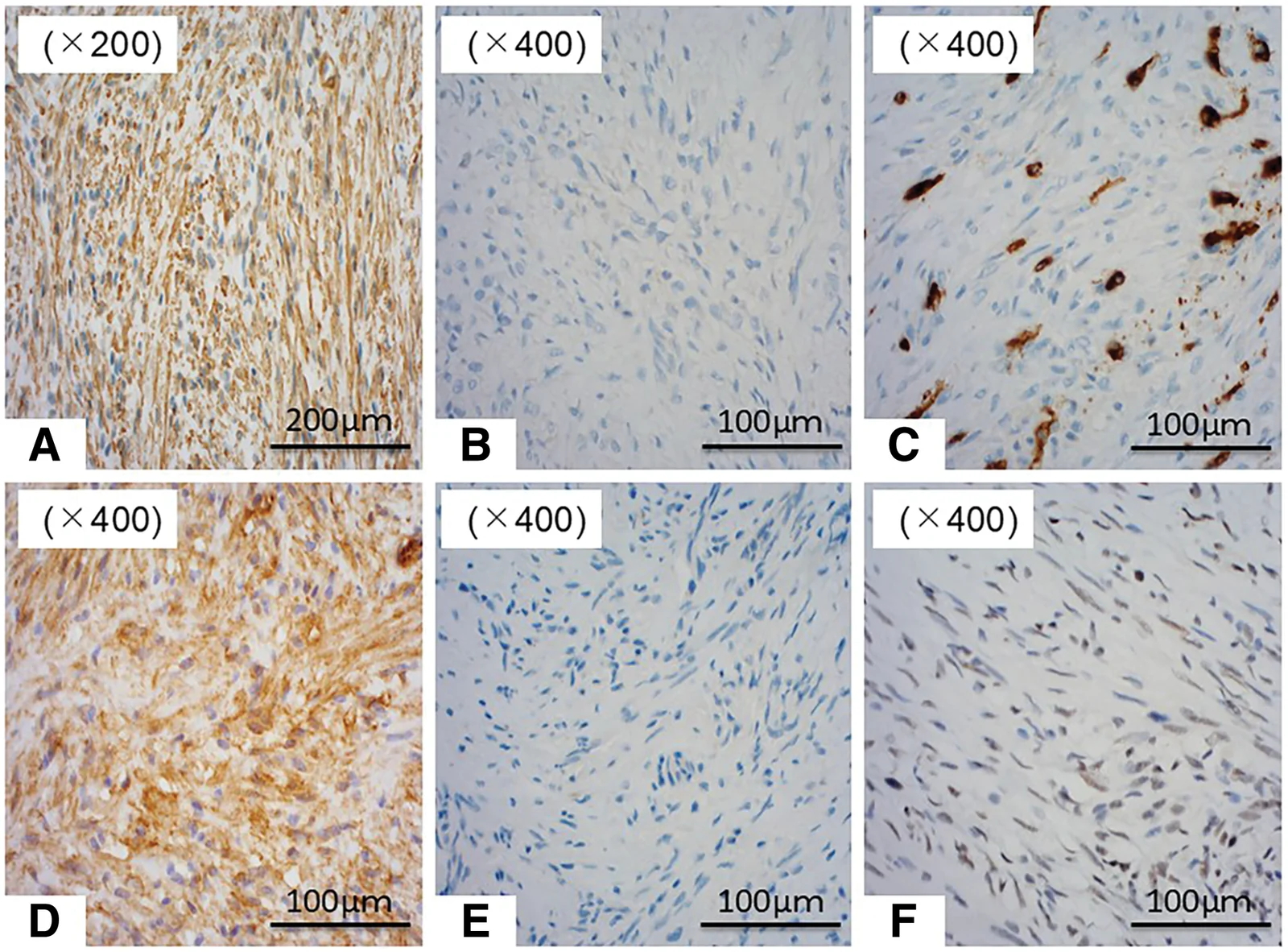
IHC analysis. (**A**) Tumor cells show diffuse cytoplasmic positivity for α-SMA (×200). (**B**) Tumor cells are negative for STAT6 (×400). (**C**) Tumor cells are negative for CD34, and endothelial cells serve as internal positive controls (×400). (**D**) β-Catenin shows cytoplasmic staining without nuclear accumulation (×400). (**E**) Tumor cells are negative for c-kit (×400). (**F**) Nuclear expression of BAP1 and expression of MTAP are retained (×400). α-SMA, alpha-smooth muscle actin; BAP1, BRCA1-associated protein 1; CD34, cluster of differentiation 34; IHC, immunohistochemical; MTAP, methylthioadenosine phosphorylase; STAT6, signal transducer and activator of transcription 6

## DISCUSSION

Previous reports have described these tumors as pleural, extrapleural, or intercostal leiomyomas according to their presumed anatomical origin. In the present report, we use the term primary chest wall leiomyoma because the exact site of origin could not be determined. Primary leiomyomas of the chest wall are rare, and only a few cases have been reported in the literature.^1)^ Leiomyomas are benign tumors originating from smooth muscle and commonly occur in the uterus or gastrointestinal tract; however, their primary occurrence in the chest wall is extremely rare. These tumors are thought to arise from microvessels in the extrapleural connective tissue and are often incidentally detected as asymptomatic lesions.^2)^ Ullah et al. reviewed cases of chest wall leiomyomas arising in the extrapleural space and reported that only 14 cases have been documented to date.^2)^

Chest wall tumors can arise from various tissues and include both benign and malignant entities, resulting in a broad range of differential diagnoses, particularly for spindle cell tumors, including malignant mesothelioma, solitary fibrous tumors, desmoid-type fibromatosis, and schwannoma.^3)^

IHC markers such as calretinin and Wilms tumor 1 for malignant mesothelioma, STAT6 and CD34 for solitary fibrous tumors, and nuclear expression of β-catenin for desmoid-type fibromatosis are useful for diagnosis.^3)^ These tumors can be morphologically similar, making a definitive diagnosis difficult based on H&E staining alone. In this case, we performed a thorough IHC evaluation to exclude these differential diagnoses and establish a diagnosis of leiomyoma. Although desmin was negative, the tumor showed positive staining for α-SMA and h-caldesmon, supporting smooth muscle differentiation. In addition, the absence of mitotic activity, infiltrative growth, and significant cytological atypia favored a benign smooth muscle tumor. Furthermore, FDG-PET showed intense uptake with an SUVmax of 11.8, which initially raised suspicion for malignancy. Although high FDG uptake is generally considered indicative of malignancy, it is difficult to distinguish between benign and malignant lesions based on SUV values alone.^4)^ High FDG uptake has been reported in a primary chest wall leiomyoma. In the previously reported case, the increased FDG uptake was attributed to concomitant osteomyelitis.^1)^ Given the patient’s history of asbestos exposure, malignant mesothelioma remained an important differential diagnosis; however, neither imaging nor intraoperative findings demonstrated definitive invasive features, and malignancy could not be ruled out. The intraoperative frozen section examination identified a spindle cell tumor but did not provide a definitive diagnosis. Therefore, we opted against extended resection, including rib resection, and decided to determine the treatment strategy based on the final pathological diagnosis. Resection with rib preservation was deemed sufficient. This case highlights that even lesions with high FDG uptake (SUVmax 11.8) can be benign and that surgical strategy should be determined based on comprehensive intraoperative and pathological evaluation. Complete resection is the standard treatment for benign chest wall tumors, with the surgical approach selected based on the tumor’s location and its relationship to surrounding structures.^3)^ In the present case, complete resection was achieved, and no recurrence was observed at the 1-year follow-up. Previous reports have indicated that primary chest wall leiomyomas typically show favorable outcomes after complete resection, with recurrence or malignant transformation being rare. However, local recurrence has also been reported in the literature.^1,2,5)^ Because the number of reported cases is limited and the long-term prognosis has not been sufficiently studied, careful long-term follow-up is necessary.

## CONCLUSIONS

Primary chest wall leiomyoma is a rare tumor that is difficult to distinguish from malignancy when it demonstrates high FDG uptake. Clinical decisions should be based on a comprehensive assessment of intraoperative findings and pathological evaluation. This case report demonstrates a successful surgical approach that avoids excessive resection.

## DECLARATIONS

### Use of artificial intelligence tools

Chat GPT (OpenAI, San Francisco, CA, USA) was used during manuscript preparation. The authors reviewed and approved the final manuscript.

### Availability of data and materials

The authors declare that all data in this article are available upon request. The data supporting the findings of this study are available from the corresponding author upon request.
